# Nutritional Properties of Edible Flowers from Five Pumpkin (*Cucurbita* sp.) Species

**DOI:** 10.3390/foods15020219

**Published:** 2026-01-08

**Authors:** Małgorzata Stryjecka, Monika Jaroszuk-Sierocińska, Anna Kiełtyka-Dadasiewicz, Barbara Krochmal-Marczak, Tomasz Cebulak

**Affiliations:** 1 Institute of Human Nutrition and Agriculture, The University College of Applied Sciences in Chełm, 22-100 Chełm, Poland; mstryjecka@panschelm.edu.pl; 2 Institute of Soil Science, Environment Engineering and Management, University of Life Sciences, 20-950 Lublin, Poland; 3 Department of Plant Production Technology and Commodity Sciences, University of Life Sciences, 20-950 Lublin, Poland; anna.kieltyka-dadasiewicz@up.edu.pl; 4 Department of Plant Production and Food Safety, The University College of Applied Sciences in Krosno, 38-400 Krosno, Poland; barbara.marczak@pans.krosno.pl; 5 Department of Food Technology and Human Nutrition, Institute of Food Technology and Nutrition, College of Natural Sciences, University of Rzeszów, 35-601 Rzeszów, Poland; tcebulak@ur.edu.pl

**Keywords:** edible flowers, nutrients, amino acids, fatty acids, vitamins, minerals

## Abstract

Edible pumpkin flowers represent a promising but still underutilized source of nutrients and bioactive compounds. Despite their traditional culinary use in various regions of the world, comprehensive studies comparing the nutritional and chemical composition of flowers from different Cucurbita species are limited. This study conducted a detailed chemical analysis of flowers from five pumpkin species: *Cucurbita maxima* (giant pumpkin), *C. pepo* (summer squash), *C. moschata* (butternut squash), *C. ficifolia* (fig-leaf gourd), and *C. argyrosperma* (cushaw squash). The analyses included the determination of basic nutritional components, amino acids, minerals, vitamins, and fatty acid profiles using standard analytical methods (AOAC, ISO, and HPLC). Significant interspecific differences were observed. The flowers of butternut squash exhibited the highest protein and fat contents, while the flowers of cushaw squash contained the largest amounts of dietary fiber and total sugars. Flowers of giant pumpkin were distinguished by their elevated contents of vitamin C and β-carotene. Amino acid analysis revealed a rich protein profile, particularly in cushaw squash, characterized by high lysine and cysteine levels, whereas fig-leaf gourd contained the greatest amounts of leucine and isoleucine. The fatty acid composition was dominated by oleic, stearic, and myristic acids, while a considerable proportion of linoleic acid (PUFA) indicated potential health benefits, such as anti-inflammatory effects. Mineral analysis showed that giant pumpkin was richest in potassium, summer squash in zinc, and butternut squash in calcium and sodium. The findings confirm that pumpkin flowers are a valuable source of nutrients and bioactive compounds. Their composition highlights their potential as functional food ingredients and as raw materials for use in the dietary, food, and pharmaceutical industries. Further studies on bioavailability and antioxidant capacity are recommended to better define their nutritional and functional value.

## 1. Introduction

The genus *Cucurbita*, belonging to the family *Cucurbitaceae*, comprises numerous cultivated plant species of economic, culinary, and medicinal importance. Among them, *Cucurbita maxima* (giant pumpkin), *C. pepo* (summer squash), *C. moschata* (butternut squash), *C. ficifolia* (fig-leaf gourd), and *C. argyrosperma* (cushaw squash) are particularly significant. These species are widely distributed around the world, occurring in both temperate and tropical regions [1,2]. Traditionally, these plants have been valued primarily for their seeds and fruits; however, in recent years, increasing attention has been directed toward their less-exploited parts, such as flowers. Pumpkin flowers not only play a crucial role in plant reproduction but also exhibit nutritional value and functional potential, placing them at the forefront of phytochemical and nutritional research [3].

Edible flowers have become an increasingly popular subject of scientific research and culinary practice due to their sensory attractiveness and potential health benefits. Several studies have demonstrated that certain flower species contain valuable bioactive compounds such as polyphenols, flavonoids, carotenoids, vitamins, and mineral elements [4]. Pumpkin flowers, traditionally consumed in Latin American and South Asian cuisines, are rich in nutrients and characterized by their delicate flavor and high digestibility [5]. Nevertheless, data concerning their complete chemical composition remain limited, particularly in terms of interspecific comparisons within the *Cucurbita* genus.

The majority of previous research has focused on the composition of pumpkin fruits and seeds, particularly with regard to their oil, protein, and mineral contents [6,7]. Pumpkin seeds are known to be a rich source of essential unsaturated fatty acids, B-group vitamins, iron, zinc, and magnesium [8]. In contrast, pumpkin flowers remain a relatively neglected research subject, even though early reports have indicated their high antioxidant potential and the presence of flavonoids such as quercetin, apigenin, and luteolin [9].

Given the growing global interest in functional foods and natural sources of bioactive compounds, there is a need for a more detailed investigation of the chemical composition of edible plant parts that have thus far been underappreciated. Pumpkin flowers, a byproduct of fruit production, may represent a valuable dietary supplement and a raw material for the development of dietary supplements, plant extracts, as well as cosmetic and pharmaceutical products [10].

The present study provides comprehensive information on the content of basic nutrients—such as proteins (including amino acids), lipids, carbohydrates, and dietary fiber—as well as vitamins and mineral components, allowing for the selection of pumpkin flower species best suited for specific dietary applications.

Therefore, a detailed chemical composition analysis of flowers from five pumpkin species—giant pumpkin, summer squash, butternut squash, fig-leaf gourd, and cushaw squash—was conducted. The study included the determination of mineral content and selected organic compounds, such as amino acids, to evaluate the nutritional and functional properties of these flowers in the context of their potential applications in the food and herbal industries.

Interspecific differences in chemical composition may result not only from genetic factors but also from agronomic conditions, microclimate, flower developmental stage, and soil type [11]. In this study, flowers of five *Cucurbita* species cultivated under identical environmental conditions were compared, thereby eliminating potential variations caused by differences in growth conditions. These findings enable the identification of the richest sources of selected compounds and may serve as a basis for future research focused on selecting *Cucurbita* varieties with enhanced biological and nutritional value.

## 2. Materials and Methods

### 2.1. Plant Material

The plant material used in this study consisted of flowers from five *Cucurbita* species: giant pumpkin (*C. maxima*) ‘Bambino’, summer squash (*C. pepo*) ‘Kamo Kamo’, butternut squash (*C. moschata*) ‘Butternut’, fig-leaf gourd (*C. ficifolia*) ‘Chilacayote Squash’, and cushaw squash (*C. argyrosperma*) ‘Chinese Alphabet’.

The plant material was obtained from crops cultivated under identical environmental conditions, as described previously [2]. From each species, 40 flowers were collected in beginning phase of flowering (60–64 BBCH) in July during the morning hours to ensure comparable physiological maturity and freshness. The collected flowers were placed in perforated paper bags and transported to the laboratory within one hour of harvesting to minimize post-harvest biochemical changes.

### 2.2. Chemical Composition Analysis (Moisture, Ash, Fat, Protein, Fiber, Carbohydrates, and Total Sugars)

Moisture content was determined using the air oven method according to AOAC 925.10 (2022) [12] by drying the samples at 105 °C to constant weight. Total ash content was measured gravimetrically following AOAC 942.05 (2023) [13] by incinerating the samples in a muffle furnace at 550 °C until white or light gray ash was obtained.

Total protein content was determined by the Kjeldahl method [14]. The nitrogen content was converted to protein using a conversion factor of 6.25.

Crude fat was extracted using the Soxhlet method [15], with petroleum ether as the solvent.

Crude fiber content was determined using the enzymatic–gravimetric method according to AOAC 991.43 (2022) [16]. After sequential hydrolysis with sulfuric acid and sodium hydroxide, the residue was weighed and subsequently incinerated in a muffle furnace to calculate the actual fiber content.

Total carbohydrate content was calculated by difference, subtracting the sum of moisture, ash, protein, fat, and fiber contents from 100%.

Total sugars were determined after the removal of proteins from the methanolic extract by precipitation with basic lead acetate. Following centrifugation and removal of the precipitate, carbohydrates were quantified in the supernatant using the anthrone method described by Yemm and Wills [17].

For the determination, 1 mL of extract was mixed with 5 mL of anthrone reagent (0.12 g anthrone dissolved in 100 mL of 6.5 M H_2_SO_4_) and incubated at 90 °C for 10 min. After incubation, samples were cooled to room temperature, and absorbance was measured at 630 nm using a UV2600i-plus spectrophotometer (Shimadzu, Kyoto, Japan).

Carbohydrate concentration was calculated based on a calibration curve prepared from glucose standard solutions, and the results were expressed as grams of glucose equivalents (GE) per unit of fresh weight. All analyses were performed in triplicate (*n* = 3), and results are presented as mean values.

### 2.3. Amino Acid Analysis

The amino acid composition was determined according to the method by Jeannerod et al. [18], with minor modifications, including adjusting the sample mass to 10 mg, reducing the hydrolysis volume (5 mL of 6 M HCl), shortening derivatization to 2 min, and slightly modifying the HPLC gradient to optimize separation. In additionhe tube was purged with nitrogen to remove air, sealed, and hydrolysed at 110 °C for 24 h. After hydrolysis, the samples were cooled, and the hydrochloric acid was evaporated under a stream of nitrogen. The residues were dissolved in 1 mL of phosphate buffer. Derivatization was then performed by adding 50 µL of ortho-phthalaldehyde (OPA) reagent to 10 µL of the sample, mixing thoroughly, and allowing the reaction to proceed for 2 min at room temperature.

The derivatized samples were analyzed using high-performance liquid chromatography (HPLC). Separation was achieved on a ZORBAX Eclipse AAA C18 column (150 × 4.6 mm, 5 µm; Agilent Technologies, Santa Clara, CA, USA) at 30 °C. The mobile phase consisted of eluent A (20 mM phosphate buffer, pH 7.2) and eluent B (acetonitrile:methanol:water, 45:45:10, *v*/*v*/*v*), with a linear gradient from 0% to 100% B over 30 min at a flow rate of 1.0 mL·min^−1^.

Fluorescence detection was used with excitation and emission wavelengths set at 340 nm and 455 nm, respectively. The injection volume was 10 µL. Quantification of individual amino acids was performed using external standards, and the results were expressed as mg of amino acid per g of protein.

### 2.4. Fatty Acid Composition Analysis

#### 2.4.1. Lipid Extraction

Total lipids were extracted using the Soxhlet extraction method with petroleum ether as the solvent, following AOAC 920.39 [19]. The obtained lipid extracts were evaporated under reduced pressure at 40 °C using a rotary evaporator (Büchi, Flawil, Switzerland). The residues were stored under a nitrogen atmosphere at −20 °C until further analysis.

#### 2.4.2. Preparation of Fatty Acid Methyl Esters (FAMEs)

Fatty acid methyl esters (FAMEs) were prepared by transesterification using methanolic potassium hydroxide according to ISO 5509:2000 [20]. Briefly, 50 mg of the extracted fat was mixed with 2 mL of 0.5 M KOH in methanol and 2 mL of hexane. The mixture was vigorously shaken for 2 min and centrifuged. The upper hexane layer containing FAMEs was collected for gas chromatographic analysis.

#### 2.4.3. Gas Chromatography (GC) Analysis

Qualitative and quantitative analyses of FAMEs were performed using gas chromatography with a flame ionization detector (GC-FID) on an Agilent 7890A chromatograph (Agilent Technologies, Santa Clara, CA, USA) equipped with a capillary column HP-88 (100 m × 0.25 mm, 0.20 μm film thickness). The operating conditions were as follows:

• Injector temperature: 250 °C; split ratio: 50:1.
• Carrier gas: helium at 1.0 mL·min^−1^.
• Temperature program: initial temperature 120 °C (held for 1 min), increased at 10 °C·min^−1^ to 175 °C (held for 10 min), then increased at 5 °C·min^−1^ to 210 °C and held for 5 min.
• Detector temperature: 260 °C.

Individual fatty acids were identified by comparing their retention times with those of a standard mixture of methyl esters (Supelco 37 FAME Mix, Sigma-Aldrich, St. Louis, MO, USA). Quantification was performed using the peak area normalization method, and the results were expressed as the percentage contribution of each fatty acid to the total lipid fraction.

### 2.5. Determination of Vitamins

#### 2.5.1. Determination of Vitamin C (Ascorbic Acid)

Vitamin C content was determined according to the method described by Tarrago-Trani et al. [21], with slight modifications. Approximately 5 g of freeze-dried pumpkin flower sample was weighed into an Erlenmeyer flask, and 50 mL of a 3% metaphosphoric acid solution was added. The mixture was extracted for 15 min in an ultrasonic bath (20 °C) and then centrifuged for 10 min at 6000 rpm. The obtained supernatant was filtered through a 0.45 μm membrane filter.

The chromatographic separation was performed using high-performance liquid chromatography (HPLC) on a Shimadzu Advanced Prominence-i system (Shimadzu, Kyoto, Japan), equipped with a ZORBAX Eclipse Plus C18 column (250 × 4.6 mm, 5 µm; Agilent Technologies). The mobile phase consisted of 0.1 M phosphoric acid, operating in isocratic mode at a flow rate of 1.0 mL·min^−1^. The column temperature was maintained at 25 °C, and the detection wavelength was set at 245 nm. The retention time for ascorbic acid was approximately 5 min. Results were expressed as mg 100 g^−1^ dry weight (DW), based on a standard calibration curve prepared from L-ascorbic acid solutions.

#### 2.5.2. Determination of B-Group Vitamins (B_1_, B_2_, B_3_, B_6_, and B_9_)

The content of B-group vitamins (thiamine, riboflavin, niacin, pyridoxine, and folic acid) was determined according to the method of Sami et al. (2014) [22], with minor modifications adapted to laboratory conditions. Approximately 5 g of freeze-dried sample was homogenized with 25 mL of phosphate buffer (0.1 M, pH 6.0). One milliliter of protease solution was added to hydrolyze the bound forms of vitamins. The mixture was incubated at 37 °C for 2 h with continuous agitation using an orbital shaker (SHHD1619AL, OHAUS, Nänikon, Switzerland).

After incubation, the enzyme was denatured by heating at 100 °C for 5 min, followed by cooling and centrifugation (10,000 rpm, 10 min, 4 °C). The supernatant was filtered through a 0.45 μm filter prior to HPLC analysis.

Chromatographic separation was performed using a Shimadzu Advanced Prominence-i HPLC system with a ZORBAX Eclipse Plus C18 column (250 × 4.6 mm, 5 µm; Agilent Technologies). The mobile phase consisted of eluent A (0.02 M sodium acetate with 5% methanol, pH 4.5) and eluent B (pure methanol, HPLC-grade). A gradient elution was applied as follows: 0–5 min, 95% A/5% B; 5–20 min, linear gradient to 50% B; 20–25 min, 50% B; then re-equilibration to initial conditions.

The flow rate was 1.0 mL·min^−1^, and the column temperature was maintained at 30 °C. The injection volume was 20 µL. Detection wavelengths were set as follows: thiamine (B_1_): 246 nm, riboflavin (B_2_): 266 nm, niacin (B_3_): 261 nm, pyridoxine (B_6_): 290 nm, and folic acid (B_9_): 280 nm. Calibration curves were constructed for each analyte in the concentration range of 0.1–10 µg·mL^−1^. All analyses were performed in triplicate. Results were expressed as mg 100 g^−1^ dry weight (DW).

#### 2.5.3. Determination of β-Carotene

β-Carotene content was determined as described by Sami et al. [22], with modifications. Two grams of freeze-dried sample were weighed into an Erlenmeyer flask, and 20 mL of an acetone–hexane mixture (2:3, *v*/*v*) containing 0.1% butylated hydroxytoluene (BHT) was added. Extraction was performed in an ultrasonic bath for 30 min in the dark. The combined extracts were filtered and evaporated under a nitrogen stream, and the residue was re-dissolved in hexane and filtered through a 0.45 µm membrane filter.

The chromatographic separation was carried out using HPLC (Shimadzu, Kyoto, Japan) equipped with a ZORBAX Eclipse XDB-C18 column (250 × 4.6 mm, 5 µm; Agilent Technologies). The mobile phase consisted of acetonitrile:dichloromethane–methanol (70:20:10, *v*/*v*/*v*) at a flow rate of 1.5 mL·min^−1^. Detection was performed at 450 nm. Quantification was based on a calibration curve prepared from β-carotene standards in the range of 0.1–10 µg·mL^−1^.

### 2.6. Determination of Mineral Components

Approximately 0.25 g of the analyzed sample was accurately weighed and subjected to microwave-assisted digestion using a Milestone ETHOS EASY system (Milestone Srl, Milan, Italy). The digestion was performed in closed Teflon vessels with 5 mL of concentrated nitric acid (HNO_3_, 65%) and 2 mL of hydrogen peroxide (H_2_O_2_, 30%) according to the manufacturer’s recommended program (temperature ramp to 180–200 °C with a holding time of 10–15 min) [23].

After digestion, the resulting solutions were diluted to 25 mL with deionized water, filtered through Whatman No. 42 filter paper, and analyzed for elemental composition using inductively coupled plasma optical emission spectrometry (ICP–OES, ICPE-9800, Shimadzu, Kyoto, Japan). Calibration was performed using external multi-element standard solutions (Merck CertiPUR, Darmstadt, Germany).

The accuracy of the analytical procedure was verified using the certified reference material NIST SRM 1573a (Tomato Leaves), with recoveries ranging from 92% to 106%.

### 2.7. Statistical Analysis

All analyses were performed in triplicate. The results are expressed as mean values ± standard deviation (SD). Statistical significance among the studied variables was evaluated using analysis of variance (ANOVA), and mean comparisons were carried out using Tukey’s honestly significant difference (HSD) test at a significance level of *p* < 0.05. All statistical analyses were performed using Statistica 13.0 software (StatSoft, Krakow, Poland).

## 3. Results and Discussion

### 3.1. Proximate Composition of Fresh Pumpkin Flowers

Table 1 presents the moisture, ash, protein, fat, carbohydrate, fiber, and total sugar contents in fresh flowers of the five analyzed *Cucurbita* species: giant pumpkin, summer squash, butternut squash, fig-leaf gourd, and cushaw squash.

The biggest moisture content was recorded in butternut squash flowers (85.32 g 100 g^−1^ FW), while the lowest was found in cushaw squash (81.97 g 100 g^−1^ FW). Regarding ash content, the highest level was observed in fig-leaf gourd flowers (1.96 g 100 g^−1^ FW), and the lowest in summer squash (0.94 g 100 g^−1^ FW). These results are higher than those observed by Kar et al. [24], who found that the moisture content of giant pumpkin flowers was 79.42% and may vary depending on the species, stage of development and environmental conditions.

The protein content observed in pumpkin flowers in this study (2.04–2.17 g 100 g^−1^ FW) was higher than the results reported by Bieżanowska-Kopeć et al. [25], who obtained a protein content of 1.14–1.50 g 100 g^−1^ FW in pumpkin flowers. The same fat content as in this study (0.15 100 g^−1^ FW) was reported by Ghosh and Rana [26] for giant pumpkin flowers. These comparisons confirm that the composition of Cucurbita flowers remains within a relatively narrow range depending on the species.

In terms of lipid content, butternut squash flowers exhibited the highest fat concentration (0.28 g 100 g^−1^ FW), while the lowest was found in giant pumpkin flowers (0.15 g 100 g^−1^ FW). Although present in small amounts, the lipids in pumpkin flowers may include beneficial unsaturated fatty acids [26]. In previous studies on giant pumpkin (‘Ambili’ cultivars), oleic and myristic acids were identified as the dominant fatty acids.

The most fiber content was found in cushaw squash flowers (0.98 g 100 g^−1^ FW), which may have positive effects on intestinal health and glucose metabolism. Although the differences between species were relatively small, the results confirm the functional character of these flowers, in agreement with the findings of Stryjecka et al. [27].

The fiber content results (0.85–0.98 g · 100 g^−1^ FW) shown in this study are within the range of values previously reported for pumpkin flowers and other edible flowers. Similar proximate values (e.g., carbohydrates ≈ 3.8 g · 100 g^−1^ FW) and low fiber values in fresh weight were reported by Bieżanowska-Kopeć et al. [25]. USDA FoodData Central reports total dietary fiber ≈ 0.9 g·100 g^−1^ for cooked pumpkin flowers, while regional studies [26] report comparable ranges for different *Cucurbita* varieties, confirming the stability of the observed fiber content in pumpkin flowers.

Carbohydrate contents were similar among all studied species, ranging from 4.18 to 4.30 g 100 g^−1^ FW. The greatest interspecific variation was observed in total sugar content: cushaw squash flowers had the highest value (2.45 g 100 g^−1^ FW), while butternut squash showed the lowest (1.16 g 100 g^−1^ FW). These variations may influence flavor properties -cushaw squash flowers may be sweeter, enhancing their culinary appeal [28].

Although the differences were relatively small, the higher fiber levels observed in cushaw squash flowers align with previous findings showing that edible flowers typically provide modest amounts of dietary fiber, which may support digestive function and help regulate postprandial glycemia [4,10].

### 3.2. Amino Acid Profile of Pumpkin Flowers

Distinct differences were observed in the amino acid composition among the analyzed *Cucurbita* species (Table 2). The predominant amino acid was glutamic acid, with the biggest concentration found in giant pumpkin (27.9 mg g^−1^ protein) and the lowest in summer squash (24.16 mg g^−1^). Aspartic acid, the second most abundant amino acid, was present in the greatest amount in cushaw squash (14.34 mg g^−1^) and the lowest in summer squash (8.17 mg g^−1^).

The content of essential amino acids also varied considerably among the studied species. The most levels of leucine (8.90 mg g^−1^) and isoleucine (4.70 mg g^−1^) were recorded in fig-leaf gourd. Cushaw squash contained the highest lysine concentration (7.45 mg g^−1^), suggesting a higher nutritional value compared to the other species.

Special attention should be paid to the sulfur-containing amino acids, cysteine and methionine, which often limit the biological value of plant proteins. The biggest cysteine content was observed in cushaw squash (1.50 mg g^−1^), whereas the highest methionine content was recorded in summer squash (1.94 mg g^−1^).

The obtained results indicate significant interspecific variation in amino acid composition, which may arise from genotypic differences among *Cucurbita* species [29]. Particularly noteworthy is cushaw squash, which exhibited the largest concentrations of several essential amino acids, including lysine and cysteine, thereby enhancing its biological value as a plant-based protein source [30].

The high levels of glutamic and aspartic acids found in all analyzed samples are typical of plant proteins [31]. In addition to its structural role, glutamic acid also functions in metabolism and neurotransmission, making it an important dietary component [32].

The well-balanced amino acid profile of fig-leaf gourd—characterized by high levels of leucine, isoleucine, and threonine—makes this species particularly attractive for use in the food and nutritional industries, especially in vegan and vegetarian diets, where sources of complete protein are limited [33].

Differences in cysteine and methionine levels may affect the indicators of protein quality, particularly the Protein Digestibility Corrected Amino Acid Score (PDCAAS). Their lower concentrations could limit the suitability of certain species as sole sources of dietary protein [34].

### 3.3. Mineral Components

In this study, the mineral composition of flowers from five *Cucurbita* species—giant pumpkin, summer squash, butternut squash, fig-leaf gourd, and cushaw squash—was analyzed. Seven elements were determined: zinc (Zn), iron (Fe), potassium (K), manganese (Mn), magnesium (Mg), calcium (Ca), and sodium (Na).

The most zinc content was observed in summer squash flowers (1.21 mg 100 g^−1^ FW), while the lowest was found in cushaw squash (0.64 mg 100 g^−1^ FW)—Table 3. Iron content ranged from 0.98 mg 100 g^−1^ in fig-leaf gourd to 1.90 mg 100 g^−1^ in giant pumpkin. Potassium levels were highest in giant pumpkin (372.63 mg 100 g^−1^ FW) and lowest in fig-leaf gourd (187.15 mg 100 g^−1^ FW).

Manganese showed the largest concentrations in cushaw squash (0.33 mg 100 g^−1^ FW) and butternut squash (0.31 mg 100 g^−1^ FW), whereas the lowest level was detected in fig-leaf gourd (0.17 mg 100 g^−1^ FW). Magnesium reached its highest value in fig-leaf gourd (29.12 mg 100 g^−1^ FW) and the lowest in cushaw squash (20.37 mg 100 g^−1^ FW). Regarding calcium, the biggest content was found in butternut squash (27.31 mg 100 g^−1^ FW), while fig-leaf gourd exhibited the lowest level (21.11 mg 100 g^−1^ FW). Sodium concentrations were greatest in butternut squash (11.35 mg 100 g^−1^ FW) and lowest in cushaw squash (8.18 mg 100 g^−1^ FW).

The obtained results indicate significant differences in mineral composition among the analyzed *Cucurbita* species, which may result from genetic factors, because the plants grew in identical conditions. White et al. [11] report that soil variability, climatic conditions, and cultivation practices may also be the reasons for the variation in mineral content. The zinc content in summer squash was the highest among all studied species, consistent with previous findings showing that pumpkin flowers can serve as a good source of this element [35]. Zinc plays an essential role in immune system function and numerous enzymatic processes [36].

The high potassium concentration in giant pumpkin (372.63 mg 100 g^−1^ FW) highlights the potential of this species as a dietary component for individuals with hypertension, considering the well-established role of potassium in blood pressure regulation [37]. Meanwhile, the elevated magnesium content in fig-leaf gourd may offer metabolic benefits, particularly in preventing metabolic syndrome and type 2 diabetes, as magnesium acts as a cofactor in over 300 enzymatic reactions, including those involved in glucose metabolism [38].

The calcium content was largest in butternut squash, suggesting that it could serve as a beneficial dietary component for populations at risk of osteoporosis, especially where dairy intake is low [39]. However, the relatively high sodium content in butternut squash is less desirable and may limit its dietary use in individuals with hypertension [40].

Cushaw squash showed lower levels of several minerals, it exhibited the highest manganese content, an essential trace element required for the activity of antioxidant enzymes such as superoxide dismutase [41].

Although ash is commonly interpreted as an estimate of total mineral matter, it is not chemically equivalent to the sum of nutritionally relevant minerals. Ash obtained by incineration includes all inorganic residues, such as oxides, carbonates, phosphates, silicates, and acid-insoluble components, as well as soil-derived particles, whereas analytical determination of minerals (ICP-OES/FAAS) captures only selected macro- and microelements. This explains why the total ash content is consistently higher than the quantified mineral sum, a phenomenon also widely reported for flower tissues and other plant materials.

### 3.4. Vitamin Content

The β-carotene content—a key precursor of vitamin A and a potent antioxidant—ranged from 2.80 mg 100 g^−1^ DW in summer squash to 3.52 mg 100 g^−1^ DW in giant pumpkin (Table 4). The biggest value observed in giant pumpkin flowers suggests a higher health-promoting potential of this species, particularly regarding the prevention of vitamin A deficiency. These findings are consistent with previous reports indicating that *Cucurbita* plants are rich in carotenoids [42].

Vitamin C content also varied considerably among the species, with the highest level recorded in giant pumpkin (25.50 mg 100 g^−1^ DW) and the lowest in summer squash (12.34 mg 100 g^−1^ DW)—Table 4. This variation suggests that genetic factors may play an important role in ascorbic acid synthesis in pumpkin flowers, as previously reported in studies comparing cultivars grown under different agronomic conditions [43].

The β-carotene concentrations measured in our study (2.80–3.52 mg 100 g^−1^ DW) were consistent with the values of 2.5–3.8 mg 100 g^−1^ DW reported for pumpkin flowers by Marchioni et al. [5]. Similarly, the vitamin C content obtained (12.3–25.5 mg 100 g^−1^ DW) closely matched the 14–26 mg 100 g^−1^ DW reported for *C. maxima* flowers grown under comparable conditions [43].

With regard to B-group vitamins, butternut squash exhibited the largest concentrations of vitamin B_1_ (0.075 mg 100 g^−1^), B_2_ (0.157 mg 100 g^−1^), B_3_ (0.782 mg 100 g^−1^), and B_6_ (0.127 mg 100 g^−1^), whereas the lowest values were recorded in fig-leaf gourd flowers (Table 4). The elevated levels of B vitamins in butternut squash may be associated with more active metabolic processes in plant tissues, as noted in previous studies [44].

Although the observed differences are not extreme, they are nutritionally meaningful—especially in the context of utilizing pumpkin flowers as a local dietary source of vitamins. Their potential application in regional cuisine or as ingredients in dietary supplements appears promising, as supported by phytochemical analyses conducted by other authors [45].

Our findings regarding B-group vitamins were also in agreement with previously published data. Butternut squash flowers contained the highest levels of thiamine, riboflavin, niacin, and pyridoxine, which is consistent with Sami et al. [22], who reported B-vitamin contents ranging from 0.03 to 0.09 mg 100 g^−1^ DW for thiamine and riboflavin and from 0.5 to 0.8 mg 100 g^−1^ DW for niacin in edible plant flowers. The numerical consistency reinforces the reliability of our results.

Overall, pumpkin flowers—particularly those of giant pumpkin and butternut squash—represent valuable natural sources of vitamins, including β-carotene and B-group vitamins. Further research is warranted to investigate the effects of cultivation conditions and flower maturity on their chemical composition, as well as to assess the bioavailability of these micronutrients.

**Table 4:** Content of water-soluble vitamins and β-carotene in flowers of five *Cucurbita* species.

Vitamins	Giant Pumpkin	Summer Squash	Butternut Squash	Fig-Leaf Gourd	Cushaw Squash
	(mg 100 g^−1^ DW)				
B1	0.053 ± 0.006 ^d^	0.035 ± 0.005 ^b^	0.075 ± 0.005 ^e^	0.027 ± 0.003 ^a^	0.037 ± 0.006 ^c^
B2	0.136 ± 0.004 ^c^	0.128 ± 0.004 ^b^	0.157 ± 0.012 ^d^	0.112 ± 0.003 ^a^	0.158 ± 0.004 ^d^
B3	0.665 ± 0.013 ^d^	0.572 ± 0.010 ^b^	0.782 ± 0.007 ^e^	0.440 ± 0.004 ^a^	0.621 ± 0.012 ^c^
B6	0.100 ± 0.002 ^c^	0.088 ± 0.004 ^a^	0.127 ± 0.004 ^e^	0.091 ± 0.003 ^b^	0.116 ± 0.005 ^d^
B9	0.057 ± 0.001 ^c^	0.054 ± 0.001 ^b^	0.066 ± 0.001 ^e^	0.041 ± 0.002 ^a^	0.062 ± 0.001 ^d^
β-carotene	3.52 ± 0.042 ^e^	2.80 ± 0.032 ^a^	3.14 ± 0.03 ^c^	3.02 ± 0.08 ^b^	3.36 ± 0.03 ^d^
C	25.50 ± 0.08 ^e^	12.34 ± 0.04 ^a^	21.31 ± 0.074 ^c^	13.59 ± 0.030 ^b^	23.50 ± 0.051 ^d^

The values in the rows marked with the same letter are not statistically significantly different at *p* < 0.05.

### 3.5. Fatty Acid Profile

Fatty acid profile analysis revealed distinct differences in the composition of individual compounds among the flowers of five *Cucurbita* species: summer squash, butternut squash, fig-leaf gourd, cushaw squash, and giant pumpkin. The dominant fatty acids across all samples were oleic acid (C18:1 n-9 cis), stearic acid (C18:0), myristic acid (C14:0), and heneicosanoic acid (C21:0)—Table 5.

The largest concentration of oleic acid was found in fig-leaf gourd (23.50%), whereas the lowest was detected in summer squash (21.63%). Myristic acid was most abundant in cushaw squash (16.93%) and least in fig-leaf gourd (15.31%). The content of heneicosanoic acid was relatively uniform, reaching its highest level in fig-leaf gourd (12.95%) and the lowest in summer squash (12.13%).

Among the saturated fatty acids (SFA), stearic acid represented a major component, ranging from 15.35% in butternut squash to 15.93% in fig-leaf gourd. Within the monounsaturated fatty acids (MUFA), oleic acid predominated, whereas among the polyunsaturated fatty acids (PUFA), linoleic acid (C18:2 n-6 cis) was the most abundant, with the biggest proportion observed in giant pumpkin (9.65%) and the lowest in summer squash (9.22%).

The content of short-chain saturated fatty acids, such as capric (C10:0) and lauric acid (C12:0), was relatively low but noticeably higher in butternut squash (2.89% and 2.91%, respectively).

The fatty acid profile of pumpkin flowers displayed both similarities and pronounced interspecific differences, likely influenced by genetic variability. The predominance of oleic and stearic acids is consistent with earlier reports on pumpkin species, where these acids constituted major components of the lipid fraction [46].

The high levels of myristic and heneicosanoic acids, particularly in cushaw squash and fig-leaf gourd, may reflect specific metabolic adaptations or variations in the enzymatic machinery involved in fatty acid biosynthesis in floral tissues. Notably, heneicosanoic acid (C21:0) is rarely found in substantial amounts in edible plant parts, and its elevated concentration may warrant further investigation into its potential biological function [47].

Conversely, the high linoleic acid content across all samples suggests potential health-promoting properties of pumpkin flowers as a source of essential polyunsaturated fatty acids. This omega-6 fatty acid plays a vital role in maintaining cell membrane integrity and regulating inflammatory responses [48].

With respect to medium-chain fatty acids (C10:0, C12:0), butternut squash exhibited the largest concentrations, which may have technological relevance—for example, in the cosmetic and pharmaceutical industries, where such acids are valued for their antimicrobial activity and high bioavailability [49].

When compared to the fatty acid profiles of other pumpkin plant parts, the composition of the flowers differed markedly from that of the seeds, which typically contain higher proportions of linoleic and oleic acids [50]. This indicates a high degree of tissue-specific lipid metabolism within the *Cucurbita* genus.

A summary analysis of the fatty acid composition in the flowers of the five *Cucurbita* species revealed a predominance of saturated fatty acids (SFA) over unsaturated ones (MUFA and PUFA). The SFA fraction ranged from 62.01% (fig-leaf gourd) to 64.49% (summer squash), highlighting the dominance of this lipid class. MUFA ranged from 26.26% (butternut squash) to 27.71% (fig-leaf gourd), while PUFA levels varied from 9.22% (summer squash) to 9.65% (giant pumpkin). The most MUFA and PUFA contents were recorded in fig-leaf gourd and giant pumpkin, respectively.

These results indicate that pumpkin flowers possess a lipid profile dominated by saturated fatty acids (SFA ~ 62–64%), which contrasts sharply with the seed lipid composition, typically enriched in polyunsaturated fatty acids, particularly linoleic (PUFA) and oleic (MUFA) acids [46]. The prevalence of SFA in floral tissues may be linked to their distinct metabolic characteristics, reflecting differences in biological function and energy requirements compared to seed tissues [50].

The contribution of MUFA (~26–28%) and PUFA (~9%) in the flowers, although noteworthy, remains relatively low compared to values typical for seed oils, such as those from summer squash seeds, where PUFA (linoleic acid) can reach 40–55% [8]. The high SFA proportion in pumpkin flowers may limit their nutritional value as a source of beneficial lipids but may simultaneously enhance oxidative stability compared to seed oils rich in PUFA [47].

From a nutritional perspective, the predominance of oleic acid (MUFA) together with the presence of linoleic acid (PUFA, n-6) is favorable, as both compounds play important metabolic and cardioprotective roles [48]. However, the relatively low PUFA content suggests that pumpkin flowers cannot be considered a significant source of essential fatty acids.

Similar observations regarding the unusual lipid profile of pumpkin flowers were reported by Ghosh and Ankaiah [26], who also found a predominance of saturated fatty acids, including myristic and stearic acids, over unsaturated ones in giant pumpkin flowers. The present findings confirm this trend while emphasizing interspecific variability—particularly the high MUFA content in fig-leaf gourd and the highest PUFA content in giant pumpkin.

### 3.6. Comparison of the Nutrition of Pumpkin Flowers with Other Edible Flowers

The nutritional profile of pumpkin flowers observed in the present study aligns with the general chemical patterns reported for other edible flowers but also demonstrates several species-specific characteristics. Similarly to nasturtium (*Tropaeolum majus*) and calendula (*Calendula officinalis*), pumpkin flowers exhibit relatively high moisture content and moderate levels of carbohydrates. However, the protein content found in butternut squash flowers was noticeably higher than values typically reported for nasturtium, hibiscus, or rose petals, which usually range between 1.2–3.5 g 100 g^−1^ FW. This indicates that pumpkin flowers, especially from butternut squash, may serve as a richer plant-based protein source compared to many ornamental edible flowers.

With respect to vitamins, the exceptionally high vitamin C concentrations found in giant pumpkin flowers exceed those reported for hibiscus (*Hibiscus sabdariffa*), rose (*Rosa rugosa*), and begonia (*Begonia cucullata*), where values generally remain within 20–60 mg 100 g^−1^ FW. Likewise, the elevated β-carotene content of pumpkin flowers is comparable to or higher than levels reported in marigold (*Calendula officinalis*), a flower known for its carotenoid-rich profile.

Mineral composition also demonstrated notable differences. Potassium levels in pumpkin flowers were higher than those reported in edible rose and chrysanthemum flowers, aligning instead with nasturtium and marigold, which are considered potassium-rich. The high calcium content in butternut squash flowers exceeds values reported for most edible flowers except orchids and certain hibiscus cultivars.

Regarding amino acids, the presence of essential amino acids such as lysine, leucine, and isoleucine in pumpkin flowers compares favorably with hibiscus, nasturtium, and elderflower, which generally contain lower concentrations of these essential amino acids.

Fatty acid profiles also distinguish pumpkin flowers from other floral species. The predominance of linoleic acid and α-linolenic acid aligns with findings in nasturtium and hibiscus, but the proportion of unsaturated fatty acids in cushaw squash flowers was higher than typically reported for marigold, rose petals, or orchids.

Taken together, these comparisons show that pumpkin flowers offer a nutrient-rich profile that complements and, in some aspects, exceeds that of commonly consumed edible flowers. Their high vitamin C, carotenoid content, favorable amino acid profile, and significant mineral levels underscore their potential as a valuable functional ingredient within the edible flower category.

The chemical composition of pumpkin flowers observed in the present study demonstrates that they represent a nutritionally valuable plant material with potential applications beyond traditional culinary uses. When compared with other edible flowers commonly used as garnishes or functional ingredients, such as nasturtium, calendula, hibiscus, rose, and begonia, pumpkin flowers—particularly those from butternut squash and giant pumpkin—display significantly higher levels of several key nutrients.

For example, the protein content identified in butternut squash flowers (2.17 g 100 g^−1^ FW) exceeds values typically reported for nasturtium, hibiscus, and rose petals, which usually range between 1.2–3.5 g 100 g^−1^ FW. This suggests that pumpkin flowers may serve as a superior botanical protein component in plant-based formulations, including soups, fillings, fermented products, and protein-enriched snacks.

The very high vitamin C and β-carotene levels observed in giant pumpkin flowers surpass those found in hibiscus, calendula, and rose flowers, positioning pumpkin flowers as a promising raw material for natural antioxidant extracts and vitamin-rich functional foods. Their high potassium and calcium contents further enhance their nutritional relevance, particularly when compared to edible flowers commonly characterized by low mineral density.

The favorable fatty acid profile, dominated by linoleic and α-linolenic acids, is comparable to that of nasturtium and hibiscus, which are frequently promoted as sources of health-promoting lipids. This suggests possible applications of pumpkin flower extracts in the development of plant-based oil blends or microencapsulated lipid supplements.

From an applied perspective, the distinctive combination of protein, vitamins, minerals, and unsaturated fatty acids supports the use of pumpkin flowers as:

• Functional ingredients in bakery, confectionery, and ready-to-eat products;
• Nutritional enhancers in soups, purees, and sauces;
• Sources of natural antioxidants in food preservation systems;
• Botanical raw materials for dietary supplements targeting micronutrient intake.

Taken together, the comparative analysis underscores the superior nutritional attributes of pumpkin flowers relative to other edible flowers and highlights their strong potential for use in both traditional cuisine and modern functional food development.

## 4. Conclusions

The evaluation of five *Cucurbita* species demonstrated that pumpkin flowers differ markedly in their nutritional and biochemical characteristics, allowing the clear identification of species with the most desirable profiles. Butternut squash flowers exhibited the highest nutritional quality, characterized by elevated protein levels, favorable essential amino-acid composition, and superior concentrations of calcium and other minerals. These features make butternut squash flowers particularly suitable for protein enrichment, mineral fortification, and incorporation into plant-based foods aimed at improving essential amino-acid balance.

Giant pumpkin flowers showed the most advantageous vitamin and antioxidant profile, with markedly higher contents of vitamin C and β-carotene compared with the other species. This highlights its potential for applications in natural antioxidant formulations, functional beverages, and micronutrient-rich dietary products.

Flowers from cushaw squash demonstrated a distinctive balance of sugars and amino acids, suggesting suitability for mildly sweet functional ingredients or formulations requiring higher essential amino-acid density. In contrast, summer squash and fig-leaf gourd showed moderate but well-balanced compositions, indicating their potential as versatile ingredients in general culinary and functional applications.

Overall, pumpkin flowers represent a nutritionally valuable and species-diverse raw material with promising applications in functional foods, plant-based product development, natural antioxidant blends, and dietary supplements. Their species-specific strengths allow targeted and purposeful use depending on the nutritional or technological goals of product formulation.

## Figures and Tables

**Table 1:** Proximate analysis of fresh pumpkin flowers from five *Cucurbita* species.

Proximate Analysis (g 100 g^−1^ FW)	Giant Pumpkin	Summer Squash	Butternut Squash	Fig-Leaf Gourd	Cushaw Squash
Moisture	84.82 ± 0.05 ^b^	83.53 ± 0.23 ^c^	85.32 ± 0.03 ^a^	82.61 ± 0.06 ^d^	81.97 ± 0.08 ^e^
Ash	1.11 ± 0.02 ^d^	0.94 ± 0.04 ^e^	1.56 ± 0.06 ^b^	1.96 ± 0.08 ^a^	1.38 ± 0.02 ^c^
Protein	2.11 ± 0.025 ^c^	2.04 ± 0.011 ^d^	2.17 ± 0.012 ^a^	2.09 ± 0.015 ^c^	2.15 ± 0.012 ^ac^
Fat	0.15 ± 0.01 ^c^	0.24 ± 0.02 ^b^	0.28 ± 0.006 ^a^	0.18 ± 0.01 ^c^	0.22 ± 0.01 ^b^
Fiber	0.90 ± 0.02 ^b^	0.97 ± 0.01 ^a^	0.85 ± 0.01 ^c^	0.94 ± 0.01 ^a^	0.98 ± 0.006 ^a^
Carbohydrate	4.20 ± 0.1 ^a^	4.30 ± 0.00 ^a^	4.18 ± 0.03 ^a^	4.24 ± 0.03 ^a^	4.02 ± 0.06 ^b^
Total sugar	1.91 ± 0.04 ^d^	2.17 ± 0.03 ^b^	1.16 ± 0.03 ^e^	2.06 ± 0.03 ^c^	2.45 ± 0.02 ^a^

The values in the rows marked with the same letter are not statistically significantly different at *p* < 0.05; FW—fresh weight.

**Table 2:** Amino acid content in the flowers of five *Cucurbita* species.

Amino Acids	Giant Pumpkin	Summer Squash	Butternut Squash	Fig-Leaf Gourd	Cushaw Squash
	(mg 100 g^−1^)				
Aspartic acid	12.88± 0.06 ^c^	8.17 ± 0.07 ^e^	12.97 ± 0.02 ^b^	11.32 ± 0.12 ^d^	14.34 ± 0.04 ^a^
Threonine	3.88 ± 0.05 ^d^	4.21 ± 0.04 ^b^	3.58 ± 0.03 ^e^	3.96 ± 0.01 ^c^	4.33 ± 0.03 ^a^
Serine	6.41 ± 0.03 ^d^	6.59 ± 0.03 ^b^	6.74 ± 0.02 ^a^	6.21 ± 0.02 ^e^	6.55 ± 0.01 ^c^
Glutamic acid	27.91 ± 0.04 ^a^	24.16 ± 0.04 ^e^	26.32 ± 0.03 ^c^	27.51 ± 0.04 ^b^	25.53 ± 0.04 ^d^
Glycine	5.91 ± 0.05 ^a^	5.76 ± 0.04 ^b^	5.65 ± 0.01 ^c^	5.58 ± 0.03 ^c^	5.49 ± 0.02 ^d^
Alanine	7.56 ± 0.05 ^c^	7.29 ± 0.05 ^d^	7.65 ± 0.01 ^b^	7.77 ± 0.02 ^a^	7.20 ± 0.03 ^e^
Cysteine	1.09 ± 0.02 ^e^	1.32 ± 0.03 ^c^	1.14 ± 0.02 ^d^	1.40 ± 0.01 ^b^	1.50 ± 0.02 ^a^
Valine	6.05 ± 0.07 ^b^	5.53 ± 0.06 ^e^	5.66 ± 0.02 ^d^	6.32 ± 0.03 ^a^	5.88 ± 0.04 ^c^
Methiionine	1.69 ± 0.04 ^c^	1.94 ± 0.02 ^a^	1.55 ± 0.02 ^d^	1.75 ± 0.01 ^b^	1.89 ± 0.02 ^a^
Isoleucine	4.22 ± 0.03 ^d^	4.56 ± 0.04 ^b^	4.39 ± 0.02 ^c^	4.70 ± 0.02 ^a^	4.59 ± 0.02 ^b^
Leucine	8.51 ± 0.02 ^c^	7.98 ± 0.04 ^e^	8.43 ± 0.03 ^d^	8.90 ± 0.04 ^a^	8.77 ± 0.03 ^b^
Tyrosine	3.41 ± 0.04 ^c^	3.54 ± 0.01 ^b^	3.42 ± 0.02 ^c^	3.79 ± 0.03 ^a^	3.38 ± 0.03 ^c^
Phenylalanine	5.45 ± 0.02 ^d^	5.14 ± 0.03 ^e^	5.63 ± 0.02 ^c^	5.84 ± 0.02 ^b^	5.94 ± 0.03 ^a^
Histydyne	4.90 ± 0.02 ^a^	4.55 ± 0.03 ^c^	4.70 ± 0.02 ^b^	4.92 ± 0.01 ^a^	4.31 ± 0.02 ^d^
Lysine	6.80 ± 0.03 ^e^	7.05 ± 0.06 ^c^	7.29 ± 0.03 ^b^	6.92 ± 0.03 ^d^	7.45 ± 0.01 ^a^
Arginine	5.25 ± 0.01 ^a^	5.05 ± 0.06 ^c^	5.15 ± 0.01 ^b^	4.92 ± 0.03 ^d^	4.65 ± 0.03 ^e^
Proline	4.89 ± 0.01 ^c^	4.96 ± 0.03 ^b^	4.70 ± 0.01 ^d^	5.14 ± 0.02 ^a^	4.58 ± 0.03 ^e^

The values in the rows marked with the same letter are not statistically significantly different at *p* < 0.05.

**Table 3:** Mineral composition of the analyzed pumpkin flowers (mg 100 g^−1^ FW).

Minerals	Giant Pumpkin	Summer Squash	Butternut Squash	Fig-Leaf Gourd	Cushaw Squash
	(mg 100 g^−1^ FW)				
Zn	0.91 ± 0.03 ^b^	1.21 ± 0.03 ^a^	0.84 ± 0.01 ^c^	0.79 ± 0.02 ^d^	0.64 ± 0.02 ^e^
Fe	1.90 ± 0.02 ^a^	1.21 ± 0.02 ^d^	1.44 ± 0.02 ^c^	0.98 ± 0.02 ^e^	1.63 ± 0.01 ^b^
K	372.63 ± 4.18 ^a^	217.07 ± 4.20 ^d^	313.07 ± 3.49 ^b^	187.15 ± 6.42 ^e^	247.42 ± 2.81 ^c^
Mn	0.28 ± 0.03 ^c^	0.23 ± 0.02 ^d^	0.31 ± 0.01 ^b^	0.17 ± 0.03 ^e^	0.33 ± 0.30 ^a^
Mg	22.59 ± 0.04 ^d^	26.40 ± 0.08 ^b^	25.53 ± 0.28 ^c^	29.12 ± 0.12 ^a^	20.37 ± 0.09 ^e^
Ca	24.21 ± 0.05 ^b^	21.14 ± 0.01 ^c^	27.31 ± 0.16 ^a^	21.11 ± 0.04 ^c^	21.18 ± 0.04 ^c^
Na	10.04 ± 0.08 ^b^	9.20 ± 0.04 ^c^	11.35 ± 0.05 ^a^	8.18 ± 0.02 ^d^	8.21 ± 0.02 ^e^

The values in the rows marked with the same letter are not statistically significantly different at *p* < 0.05.

**Table 5:** Fatty acid composition in the flowers of five *Cucurbita* species.

Fatty Acids	Giant Pumpkin	Summer Squash	Butternut Squash	Fig-Leaf Gourd	Cushaw Squash
	%				
Capric acid (C10:0)	1.32 ± 0.04 ^e^	2.17 ± 0.02 ^b^	2.89 ± 0.04 ^a^	1.80 ± 0.03 ^c^	1.54 ± 0.04 ^d^
Lauric acid C12:0)	2.62 ± 0.03 ^c^	2.24 ± 0.04 ^d^	2.91 ± 0.03 ^b^	2.18 ± 0.05 ^e^	2.95 ± 0.04 ^a^
Myristic acid (C14:0)	16.24 ± 0.06 ^d^	16.66 ± 0.02 ^b^	16.44 ± 0.02 ^c^	15.31 ± 0.06 ^e^	16.93 ± 0.06 ^a^
Palmitic acid (C16:0)	4.61 ± 0.05 ^b^	4.91 ± 0.04 ^a^	4.46 ± 0.03 ^c^	4.26 ± 0.03 ^e^	4.37 ± 0.02 ^d^
Palmitoleic acid(C16:1 n-7 cis)	4.41 ± 0.04 ^b^	4.66 ± 0.01 ^a^	4.35 ± 0.04 ^c^	4.21 ± 0.04 ^e^	4.24 ± 0.01 ^d^
Stearic acid (C18:0)	15.49 ± 0.04 ^d^	15.83 ± 0.04 ^b^	15.35 ± 0.03 ^e^	15.93 ± 0.04 ^a^	15.61 ± 0.05 ^c^
Oleic acid (C18:1 n-9 cis)	23.12 ± 0.05 ^b^	21.63 ± 0.02 ^e^	21.91 ± 0.03 ^d^	23.50 ± 0.04 ^a^	22.79 ± 0.05 ^c^
Linoleic acid (C18:2 n-6 cis)	9.65 ± 0.02 ^a^	9.22 ± 0.05 ^e^	9.44 ± 0.02 ^b^	9.28 ± 0.01 ^d^	9.36 ± 0.02 ^c^
Arachidic acid (C20:0)	6.36 ± 0.01 ^c^	6.63 ± 0.02 ^b^	6.25 ± 0.03 ^e^	6.73 ± 0.01 ^a^	6.30 ± 0.03 ^d^
Heneicosanoic acid (C21:0)	12.50 ± 0.04 ^d^	12.13 ± 0.02 ^e^	12.80 ± 0.03 ^b^	12.95 ± 0.04 ^a^	12.76 ± 0.01 ^c^
Behenic acid (C22:0)	3.68 ± 0.03 ^c^	3.92 ± 0.04 ^a^	3.20 ± 0.03 ^d^	3.85 ± 0.02 ^b^	3.15 ± 0.04 ^e^
MUFA	27.53	26.29	26.26	27.71	27.03
PUFA	9.65	9.22	9.44	9.28	9.36
SFA	62.82	64.49	64.30	62.01	63.61

The values in the rows marked with the same letter are not statistically significantly different at *p* < 0.05.

## Data Availability

The original contributions presented in this study are included in the article. Further inquiries can be directed to the corresponding author.
